# A comparative examination of tuberculosis immigration medical screening programs from selected countries with high immigration and low tuberculosis incidence rates

**DOI:** 10.1186/1471-2334-11-3

**Published:** 2011-01-04

**Authors:** Gonzalo G Alvarez, Brian Gushulak, Khaled Abu Rumman, Ekkehardt Altpeter, Daniel Chemtob, Paul Douglas, Connie Erkens, Peter Helbling, Ingrid Hamilton, Jane Jones, Alberto Matteelli, Marie-Claire Paty, Drew L Posey, Daniel Sagebiel, Erika Slump, Anders Tegnell, Elena Rodríguez Valín, Brita Askeland Winje, Edward Ellis

**Affiliations:** 1 Divisions of Respirology and Infectious Diseases, University of Ottawa at The Ottawa Hospital, The Ottawa Health Research Institute, 501 Smyth Road, Ottawa, Ontario, Canada; 2 Migration Health Consultants, Inc., Singapore; 3 National Tuberculosis Program, Jordan; 4 Eidgenössisches Departement des Innern EDI, Bundesamt für Gesundheit BAG, Direktionsbereich Öffentliche Gesundheit, Schwarztorstrasse 96, CH-3007 Bern, Switzerland; 5 National Tuberculosis Program, Department of Tuberculosis and AIDS, Public Health Services, Ministry of Health33 Pierre Koenig, P.O.B 1176, Jerusalem 91010, Israel; 6 Department of Immigration & Citizenship Building C, Level 6, 300 Elizabeth St, Sydney, Australia; 7 KNCV Tuberculosis Foundation, PO Box 146, 2501 CC The Hague, The Netherlands; 8 Communicable Diseases, Population Health Protection Group, Population Health Directorate, Ministry of Health, New Zealand; 9 Travel and Migrant Health Section, Health Protection Agency, Centre for Infections, 61 Colindale Avenue, London, England; 10 Istituto Malattie Infettive e Tropicali, Università degli Studi di Brescia, P.le Spedali Civili, 1, 25123, Italy; 11 Direction Générale de la Santé, Sous direction Prévention des risques infectieux, Bureau risque infectieux et politique vaccinale - RI 1, 14, avenue Duquesne, 75350 Paris, France; 12 CDR, USPHS, Immigrant, Refugee, and Migrant Health Branch, Division of Global Migration and Quarantine, Centers for Disease Control and Prevention, USA; 13 Department for Infectious Disease Epidemiology, Robert Koch Institute, DGZ-Ring 1, 13086 Berlin, Germany; 14 Socialstyrelsen, The National Board of Health and Welfare, Communicable Disease Prevention and Control, 10630 Stockholm, Sweden; 15 Centro Nacional de Epidemiología, Instituto de Salud Carlos III C/Sinesio Delgado n° 6, 28029-Madrid, Spain; 16 Norwegian Institute of Public Health Agency of Canada, 100 Eglantine Drive, AL 0603B Ottawa, Ontario K1A 0K9, Canada; 17 Tuberculosis Prevention and Control, Public Health Agency of Canada, 100 Eglantine Drive, AL 0603B Ottawa, Ontario K1A 0K9, Canada

## Abstract

**Background:**

Tuberculosis (TB) in migrants is an ongoing challenge in several low TB incidence countries since a large proportion of TB in these countries occurs in migrants from high incidence countries. To meet these challenges, several countries utilize TB screening programs. The programs attempt to identify and treat those with active and/or infectious stages of the disease. In addition, screening is used to identify and manage those with latent or inactive disease after arrival. Between nations, considerable variation exists in the methods used in migration-associated TB screening. The present study aimed to compare the TB immigration medical examination requirements in selected countries of high immigration and low TB incidence rates.

**Methods:**

Descriptive study of immigration TB screening programs

**Results:**

16 out of 18 eligible countries responded to the written standardized survey and phone interview. Comparisons in specific areas of TB immigration screening programs included authorities responsible for TB screening, the primary objectives of the TB screening program, the yield of detection of active TB disease, screening details and aspects of follow up for inactive pulmonary TB. No two countries had the same approach to TB screening among migrants. Important differences, common practices, common problems, evidence or lack of evidence for program specifics were noted.

**Conclusions:**

In spite of common goals, there is great diversity in the processes and practices designed to mitigate the impact of migration-associated TB among nations that screen migrants for the disease. The long-term goal in decreasing migration-related introduction of TB from high to low incidence countries remains diminishing the prevalence of the disease in those high incidence locations. In the meantime, existing or planned migration screening programs for TB can be made more efficient and evidenced based. Cooperation among countries doing research in the areas outlined in this study should facilitate the development of improved screening programs.

## Background

Medical screening related to the process of immigration commonly includes screening for tuberculosis (TB). The intent and rationale of the process is to diagnose and treat active, infectious TB before arrival, or as soon as possible after arrival, in order to prevent transmission to persons in the host country. Secondary benefits of immigration screening are reduced transmission of TB in the country of origin and during travel. Growing incidence of multi drug resistant (MDR) and even extensively drug resistant (XDR) TB in some regions of the world underscore the importance for screening of migrants. Screening for TB among migrant populations has been shown to lead to earlier detection of cases, resulting in a shorter duration of symptoms, and fewer hospitalizations. The screening may decrease the period of infectiousness by as much as 33% in some situations[1]. Additionally, the identification of those with inactive pulmonary TB (PTB) or latent TB infection (LTBI) provides opportunity for early treatment and the prevention of significant health sequelae for the individual. In spite of their benefits, TB immigration screening programs are subject to many shortcomings. It has been suggested that pre-departure screening detects only a small number of active cases[2,3], which is the primary objective of most programs. Some have contended that very few active infectious cases are identified[4] due in part to the screening tools used and programmatic challenges faced within immigration TB screening programs.

The chest radiograph (CXR) has been and remains the central tool in screening for TB among migrants in most programs. However the CXR's sensitivity and specificity for the diagnosis of active pulmonary TB is only in the range of 64-80% and 52-63% respectively [5-8] and is thus not the gold standard. The CXR alone has been reported to have a low negative predictive value for the identification of active TB in migrants[9]. Using estimates from the literature one group estimated that the positive predictive value of the CXR was less than 1% assuming a prevalence of active disease of 1%[10]. Thus reliance on CXR leads to many false positive results, unnecessary further testing, and follow up. This finding is supported by another recent study[11]. On the other hand, radiographic screening has demonstrated benefits in screening large numbers of individuals in high prevalence populations where the background level of disease may be higher[12].

The other major component of immigration-related TB screening is the referral for evaluation and possible treatment for individuals determined to have inactive PTB or LTBI detected during screening. Known by a variety of descriptions such as "post-arrival screening", "medical surveillance" or a "health undertaking," compliance with this screening component determines its effectiveness. A Canadian study[13] determined that 14% of foreign-born patients developing active PTB after arrival had been noted to have inactive PTB during their immigration medical examination. However, only 20% of patients were examined after arrival and adhered to treatment. More recent studies have indicated that an average of 50% of such individuals are now completing post-immigration medical surveillance requirements in Canada[14].

Concerns regarding the benefits and costs of immigration screening for TB have raised suggestions that there may be alternative, more effective means of dealing with TB resulting from immigration. It has been demonstrated that the risk of TB transmission to host populations from migrants is in fact low[15-17]. For this reason, some suggest the use of contact tracing of active cases in migrants as a more efficient and cost effective way[18] of managing TB among the immigrant population as opposed to routine immigrant TB screening[19].

The lack of robust evidence for TB immigration screening and significant operational problems encountered by programs[13,18] may in part explain the significant variation among national TB immigration screening programs. Some nations utilize pre-departure screening, such as Australia, Canada and the United States of America (U.S.), where international immigration represents a longstanding national policy for nation building. Other nations where large-scale immigration represents a newer social process have different practices. In one study[20], 13 (50%) of 26 European countries did not have any specific TB screening program for migrants. All of the countries that had programs principally screened refugees and asylum seekers, (known as refugee claimants in some countries). None did pre-departure screening; 3 screened at the port of entry and 9 in reception or holding centers. No two European countries took the same approach to screening. Similar diversity in practices was noted by another study[21] examining the service model delivery system of screening. The present study was designed to compare certain aspects of established national TB immigration screening programs of selected high immigration low incidence TB countries. Gaps in knowledge were identified to help direct future research in the field.

## Methods

### Country selection

Country selection was based on the following criteria (Table 1):

**Table 1:** High immigration countries with respective incidences of all forms of TB and smear positive active pulmonary TB (PTB)

Country	% contribution to world migration 2005* (in millions)†	All forms of TB incidence rate per 100,000 reported in 2006§ (absolute number of cases)	Smear positive PTB† incidence rate per 100,000 reported in 2006§ (absolute number of cases)	WHO 3 year estimate average sputum positive smear PTB incidence rate per 100,000	Included in survey
**United States**	**20.2 (38.4)**	**4 (13,148)**	**2 (5,777)**	**2**	**Yes**

Russian Fed	6.4 (12.1)	107(152,797)	48(68,178)	51	No

**Germany**	**5.3 (10.1)**	**6 (5,370)**	**3 (2,407)**	**3**	**Yes**

Ukraine	3.6 (6.8)	106(49,308)	47(21,902)	45	No

**France**	**3.4 (6.5)**	**14(8,630)**	**6 (3,830)**	**6**	**Yes**

Saudi Arabia	3.3 (6.4)	44(10,631)	20(4,784)	19	No

**Canada**	**3.2(6.1)**	**5(1,678)**	**2 (748)**	**2**	**Yes**

India	3.0(5.7)	168(1,932,852)	75(867,455)	75	No

**United Kingdom**	**2.8(5.4)**	**15(9,358)**	**7(4,177)**	**6**	**Yes**

**Spain**	**2.5(4.8)**	**30 (13,179)**	**13 (5,810)**	**12**	**Yes**

**Australia**	**2.2(4.1)**	**6(1,329)**	**3(595)**	**3**	**Yes**

Pakistan	1.7(3.3)	181(291,743)	82(131,192)	82	No

**UAE (United Arab Emirates)**	**1.7(3.2)**	**16 (681)**	**7(306)**	**7**	**Yes**

Hong Kong, SAR China	1.6(3.0)	62(4,433)	28(1,995)	32	No

**Israel**	**1.4(2.7)**	**8(521)**	**3(233)**	**3**	**Yes**

**Italy**	**1.3(2.5)**	**7(4,393)**	**3(1,945)**	**3**	**Yes**

Kazakhstan	1.3(2.5)	130(19,961)	59(8,971)	64	No

Cote d'Ivoire	1.2(2.4)	420 (79,515)	183(34,669)	173	No

**Jordan**	**1.2(2.2)**	**5 (306)**	**2 (138)**	**2**	**Yes**

**Japan**	**1.1(2.0)**	**22(28,330)**	**10(12,736)**	**12**	**Yes**

*United Nations, Trends in total migrant stock: The 2005 revision[22]

†The number of international migrants, also called the international migrant stock, generally represents the number of persons born in a country other than that in which they live.

‡ PTB - pulmonary TB

§ World Health Organization, Tuberculosis Control, Surveillance, Planning and Finances[54].

1) One of the 20 countries accepting the most immigrants based on the United Nations' *World Migration 2005 Report (published every 5 years)*[22] OR

2) Immigration medical TB screening results published in any peer-reviewed journal AND

3) A low domestic TB incidence rate defined as a rate of sputum smear positive PTB of < 15 cases per 100,000 population as estimated by the World Health Organization (WHO) averaged over the years 2004, 2005 and 2006[14].

### The survey

The survey was completed during the months of July and August 2008. Country specific details reported in this document were accurate as of April 2009 and for the US October 2009. Variables included immigration-associated TB epidemiology, authorities for TB screening, the primary objectives of the TB screening program, the yield of TB detection, operational aspects and requirements of pre departure and post arrival screening for medical or public health follow up for inactive PTB or LTBI. The information collected was focused on established national policies and guidelines rather than individual clinic procedures within nations and is publically available.

### Statistics

Stata version 10 (Statacorp, College Station, Texas, USA) software was used to calculate differences between incidence rates of TB between countries using the unpaired t-test.

## Results

### Participation in the study

Of 18 countries initially selected 16 (89%) participated in the study. This included 11 of 12 countries selected on the basis of high foreign-born populations and low domestic TB incidence. Of six countries chosen for the study on the basis of publications regarding their TB immigration screening programs, five responded. All participating countries are listed in Table 2.

**Table 2:** Country TB rates per 100,000 population for 2006

Country	Incidence of all forms of TB as reported by the country	Incidence of smear positive PTB* as reported by the country	Foreign born proportion of all cases of TB%	% of cases for which country of birth status is known†
**TB screening program present**				

United States	4.6	2‡	57	

Germany	6.6	1.7	43.3	97

France	8.5	3.3	45	

Canada	5	2	64	97

United Kingdom	15‡	7‡	73	

Australia	5.8	3	81.6	

Israel	5.5	1	82.3	

Switzerland	7	2	78	75

Jordan	6.4	2	38	

New Zealand	8.6	2.6	70	85

Netherlands	6.2	1.3	63	

Norway	6.3	1	81	

Sweden	5.5	1.2	72	

				

**Mean**	**7.0**	**2.3**	**65**	

				

**TB screening programs absent**				

Japan	22‡	10‡	4	

Spain	18.3	5	25	76

Italy	7.5	3	46.2	

				

**Mean**	**15.9**	**6.0**	**25**	

*PTB - pulmonary tuberculosis

†Number represents the percent of individuals with TB where the country of origin for the case was known

‡ World Health Organization (WHO) 2006 rates[54], country reported rates may differ from estimates prepared by the WHO which uses various epidemiological models to estimate rates.

### Immigration TB epidemiology of surveyed countries

For all of the countries studied the mean incidence rate for all forms of TB in 2006 was 8.7/100,000 (95% CI (5.9 - 11.4)) and for smear positive PTB, the mean incidence rate was 3/100,000 (95% CI (1.7 - 4.3)). Italy, Japan and Spain did not have formalized TB screening programs for migrants. A significant difference was noted between the mean incidence rates of all forms of TB, 7.0/100,000 (95% CI (4.8 - 9.2)) in countries with a screening program compared to 15.9 per 100,000 (95% CI (11.3-20.6)) in countries with no screening program (p = 0.002). A significant difference was also noted between the mean incidence of smear positive pulmonary TB of 2.3 per 100,000 (95% CI (1.1-3.5)) for countries with a screening program compared to the mean incidence of 6.0 per 100,000 (95% CI (3.5-8.5)) for countries with no screening program (p = 0.012). The foreign born constituted a mean of 58% of the TB cases reported in all of the surveyed countries. A significant difference was noted when the foreign born proportion of all cases was compared between countries that had a TB screening program and those that did not have a TB screening program (65.2, 95% CI (55.6 - 74.9), 25.1, 95% CI (4.9 - 45.19), p = 0.002).

### Objectives of the Immigration Medical Exam (IME) in relation to TB

The primary objective of all screening programs was to identify active PTB. However, in Israel, and Sweden screening included the routine detection of LTBI using tuberculin skin testing (TST). In Canada, Australia and the U.S., individuals deemed to be at greater risk of inactive PTB were identified on the basis of the CXR findings.

### Authority responsible for TB immigration medical examination (IME) screening

Funding for the screening TB program structure was provided by the government across all countries surveyed. All countries that screen for TB *in the country of origin *require the immigrant applicant to pay for any tests including the CXR. All countries surveyed, except for the United States, paid for the treatment of an active case detected through screening *in the host country*. All stated that the detection of active TB did not affect the overall outcome of the immigration application. However, the immigration process was interrupted or delayed until the individual successfully completed TB treatment if active TB was detected on screening.

### Immigration TB screening effectiveness

France, Jordan, the Netherlands and Switzerland routinely documented the yield of active PTB detected through the screening program, with rates in 2006 ranging from 153/100,000 persons screened in Jordan to a rate of 70/100,000 in France (Table 3). Australia's estimated rate in 2006 was 43/100,000 while New Zealand's was 221/100,000. While the Canadian estimate for 2006 was 101 per 100,000, precise counting of cases in 2008 resulted in a rate of 54/100,000.

**Table 3:** Number of immigrants and refugees that had TB immigration screening done, the absolute number and calculated rates of active TB cases detected in 2006*

Country	Number of immigrants and refugees screened		Absolute number of active cases detected†	Calculated number of active cases detected per 100,000	Calculated percent yield (absolute/total × 100)
	**In country**	**Out of country**			

Canada*	111,280	407,206	278	53.6	0.05%

France	205,713		143	70	0.07%

Jordan	212,428	43,414	391	153	0.15%

Netherlands	63,268		67	105	0.11%

Switzerland	8,995		11	122	0.12%

					

**Estimates**					

Australia	200,000	500,000	400	80	0.08%

New Zealand	126,213‡		279‡	221	0.22%

*2008 provisional data for Canada

† The definition of an active cases included: smear AFB positive and or culture TB positive

‡ The number represents both in and out of country migrants and those applicants who were staying over 12 months and submitted full medical. Applicants who may have been screened with only a CXR and no medical (those people from high-incidence countries who are staying for more than 6 months but less than a year) would not have been included and the 279 represents the number of people who were defined as having an unacceptable standard of health out of the people who submitted full medical certificates likely to mean they had TB however this data was not available.

### Demographic characteristics of those subject to immigration TB screening

Australia, Canada, France, the Netherlands, New Zealand, Norway, United Kingdom (U.K.) and the U.S. screen all categories of those applying for permanent residence. This includes immigrants, asylum seekers and refugees (Table 4). Germany, Sweden and Switzerland screen only asylum seekers and for Germany also re-settlers. Israel's program focuses on the geographical origin of migrants rather than the type of migrant, although any migrant who is coming for the purposes of work in Israel is screened in the country of origin.

**Table 4:** Type of migrants that get screened

Country	Permanent residency application			Temporary residency application		
	**Immigrant**	**Asylum seekers**	**Convention refugee**	**Visiting Students**	**Temporary Workers/Visitors**	**Specific**

**United States**	+	+	+	--	--	--

**Germany**	+	+	-	--	--	--

**France**	+	+	+	> 3 months	> 3 months	--

**Canada**	+	+	+	> 6 months*	> 6 months*	Occupation†

**United Kingdom**	+	+	+	> 6 months	> 6 months	--

**Australia**	+	+	+	> 1 months (>100/100K) > 3 months (> 60/100K) > 12 month (< 20/100K)‡	Nursing, dental, child care worker	Occupation†

**Israel**	--	--	--	--	+	--

**Jordan**	+	+	--	> 3 months	> 3 months	Adoptee

**Additional Countries**						

**Norway**	+	+	+	> 3 months	> 3 months	Adoptee

**New Zealand**	+	+	+	> 6months (with risk factors)** > 12 months (all)	> 6months (with risk factors)** > 12 months (all)	--

**Switzerland**	--	+	--	--	--	--

**Sweden**	--	+	--	--	--	--

**Netherlands**	+	+	+	> 3 months	> 3 months	Adoptee

**+ = YES and **-- **= NO**

*Temporary residents who will stay in Canada for more than 6 months and who have spent >6 consecutive months in a high incidence countries are also screened.

‡ In Australia, countries with TB rates < 20/100,000 get a CXR if coming for > 12 months and if ≥ 20/100,000 than a CXR is done if coming for more than 3 months >.

** New Zealand screens all applicants intending to stay for more than 12 months for TB. It also screens applicants intending to stay more than six months if there are risk factors present (ie. they hold a passport for a country not considered to be low incidence of TB, or if, in the five years prior to application, they have spent three months or more in any such country/ies)

Major differences in approach were noted among countries surveyed in regards to those arriving for temporary periods of residence (Table 4). Germany, Sweden, Switzerland, the U.K. and the U.S. do not screen temporary residency applicants. Australia, Canada, France, Israel, Jordan, New Zealand, Netherlands and Norway do screen temporary migrants who are staying longer than 3-6 months. Australia uses the country of origin TB prevalence rate to determine which temporary migrants they will screen (e.g. screen if the TB rate exceeds 100/100,000 population and the migrant will stay >1 month). In the U.S., individuals who are not immigrants or refugees, namely, students, workers, visitors and illegal migrants are not routinely screened for TB as part of the immigration screening program[23].

### Occupation

Several countries including Australia, Canada, and New Zealand screen applicants working in occupations associated with risks of work-related disease transmission. These include health care providers, teachers and childcare providers. In Canada, foreign nationals are required to submit to an immigration medical examination if they are seeking to work in an occupation in which the protection of public health is essential whatever the length of stay in Canada or country of origin. In Norway, examination is required for persons who have arrived from, or who have spent at least three months in, a country with a high prevalence of TB require pre-employment screening.

### Location of screening

Immigration screening for TB may take place in three locations:

(1) *Pre-departure screening *commonly in the country of origin

(2) *On arrival screening *commonly occurs at the point of entry into the host country and at airports and other centers were migrants transit

(3) *Post arrival screening *occurs once the migrant has left the airport or centers used for transitioning. It includes post arrival surveillance or follow up of identified cases. Post arrival screening may be active where the foreign-born are required to undergo examination because of their immigration status. Alternatively, it may by passive and undertaken during routine health care as recommended by national guidelines.

Countries receiving a high number of immigrants and having a national PTB incidence rate of < 15/100,000 population (Table 1) tended to screen applicants in the country of origin as compared to countries that did not form part of this list but were included in our study (Table 5). Countries accepting a lower number of migrants tended to screen after the migrant had arrived. Most asylum seekers were screened sometime after arrival. However, some countries utilizing reception or holding centers for those seeking asylum undertook screening immediately on arrival at the center. Australia, Canada and the U.S. screened convention refugees in the country of origin. The U.K. had several possible points of screening (pre/at/post entry) depending on country of origin, route in to the U.K. and migration status. Most survey respondents from countries that did pre-departure screening identified the lack of quality-assured health care services in other countries as a major limitation of screening abroad.

**Table 5:** Point of screening

Country	Immigrants			Refugees/Asylum seekers		
	**Country of origin**	**On arrival**	**After arrival**	**Country of origin**	**On arrival**	**After arrival**

**United States**	+	--	+	+	--	+

**Germany**	--	--	--	--	--	+

**France**	+	--	+	--	--	+

**Canada**	+	--	--	+	--	+

**United Kingdom**	+	+	+¥		+	

**Australia**	+	--	--	+	--	+

**Israel**	+*	--	--	--	--	--

**Jordan**	+†	--	+	--	--	+

**Additional Countries**						

**Norway**	--	+	+	--	+	--

**New Zealand**	+	--	+	+	-	+

**Sweden**	--	--	+	--	--	+

**Switzerland**	--	--	--	--	+	--

**Netherlands**	--	--	+	--	--	+

**+ = YES and **-- **= NO**

* Ethiopia is the only country where Israel is performing CXRs and TST (1^st ^step) for screening in the country of origin

†Jordan does screen applicants from Sri Lanka, Indonesia and the Philippines abroad

¥ For programme refugees

### Screening for active TB disease

#### Chest radiograph (CXR)

Differences among countries existed in the type of physician who interpreted the CXR. These ranged from general practitioners who are designated medical practitioners (DMP) or panel doctors to TB specialists, respirologists or radiologists. The countries that carry out pre-departure screening had systems in place for local radiologists to read the CXR. In Australia all x-rays are read by DMPs from all high risk countries (total TB incidence >100/100 000 general population ) and panel doctors from others are audited through desk top reviews by DMPs in Sydney. Thus, all panel radiologists are supervised by DMPs. Only Israel had a centralized method of reading CXRs where two radiologists read all of the immigration screening CXRs. In Canada, CXRs are examined by a local radiologist in the country of origin and then another assessment is done by a physician hired by the Canadian government to perform that task or by a Canadian Immigration Medical Officer. Canada is the only country surveyed to use a scoring system to further quantify the risk of TB disease. The 18 factor ascending scale is designed to detect active and inactive PTB[14].

#### Respiratory secretion testing

All countries indicated that they used both acid-fast bacilli (AFB) smear and culture in applicants with abnormal CXRs and/or clinical signs and symptoms of TB. Depending on country and location, cultures may be obtained from sputum, bronchial aspiration or gastric lavage. Technical instructions and guidance vary by country and these guidelines are periodically modified. For example the U.S. is in the process of implementing new TB guidelines (2007 Techncial Instructions for Tuberculosis Screening and Treatment) requiring regular collection of sputa for microscopy and culture and the routine use of TSTs for those who do not require a radiograph[24]. Historically, laboratory investigation was only required for those suspected of having active PTB. Drug susceptibility testing is now required for all positive cultures.

#### TST

The U.S. (where the 2007 TB TI has been implemented) use the TST for screening children for active TB disease. The Netherlands use the TST to screen all unvaccinated children for active TB disease.

### Screening for LTBI

#### TST

Israel, Norway and Sweden use the TST to diagnose LTBI in migrants entering the host country. While most countries do not screen for LTBI as part of the immigration screening program, they do recommend the use of the TST to screen high risk immigrants in the primary care setting after the immigration process is completed. The reasons for not using the TST [25] include: (1) false positives due to BCG (bacille Calmette-Guérin) vaccination or infection with environmental mycobacteria, (2) very large number of TST positive applicants, (40-50% of adult immigration applicants have lived for 20 years or more in high TB incidence countries[14]), (3) low reactivation risk among many with a positive TST and (4) poor adherence to current LTBI treatment regimes in most programs[26].

#### CXR for detecting inactive PTB as a form of LTBI

In Canada, inactive PTB cases are defined by a combination of radiological and laboratory criteria. In the absence of clinical symptoms, abnormalities compatible with PTB that are radiologically stable for more than 6 months are considered to represent inactive PTB. Similar conclusions result from three-month radiographic stability in the presence of negative smears and cultures[14]. In Canada and Australia, applicants who have inactive PTB detected on CXR are put on medical surveillance or receive a health undertaking to attend for review and follow up after arrival.

#### Interferon Gamma Release Assays (IGRA) for detection of LTBI

Norway and the U.S., where the 2007 TB TI are in use, are the only surveyed countries that have adopted the IGRA as part of the national immigration TB screening program. A study in Norway demonstrated that use of an IGRA would have reduced the number of asylum seekers needing further follow up by 43%[27]. Although this would not affect the number of applicants referred for screening, use of the IGRA could improve the selection of persons to refer for further evaluation.

### Post arrival immigration medical surveillance

Managing inactive PTB is an important component of the immigration screening programs of some countries. Known as medical surveillance, a health undertaking or medical referral, it involves post-arrival medical evaluation in order to consider treatment to prevent progression of inactive PTB to active disease. The process owes its origin and name to the practice of regular clinical review of TB patients to watch for disease reactivation before preventive treatment for inactive PTB was available. It does not represent epidemiological surveillance of the disease.

In Canada, inactive PTB is diagnosed in 2% of all immigration applicants[14] and associated with a five time higher risk of reactivation when compared to applicants with a normal CXR[18]. In some studies in Canada, 1.5-2.8% of persons referred for medical surveillance were diagnosed with active TB at their first medical evaluation[28,29]. Eight countries including France, Germany, Netherlands, New Zealand, Norway, Sweden, Switzerland and the U.S. did not report formal programs to review those with inactive PTB or latent infection in the post arrival phase of the immigration process (Table 6). Although the U.S. does not have a legally binding process for follow-up evaluations or surveillance of arriving persons suspected of having TB, they are in the process of fully implementing an Electronic Disease Notification (EDN) system that transmits information on arriving immigrants and refugees with a TB classification to receiving health departments. Prior to EDN, there was a paper-based system for notifying receiving health departments of arrival for all refugees and all immigrants suspected of having TB. Two countries, Australia and the Netherlands, had collected data on compliance with the first follow up visit measured at 80% and 59% respectively.

**Table 6:** Post arrival immigration surveillance

Country	Refugee Center	Follow up post arrival	Compliance with first follow up	Consequences to not coming to follow up
**United States**	No	No	--	--

**Germany‡**	Yes	No	--	--

**France**	Yes	No	--	Residency permit not issued

**Canada**	Yes	Yes	49%	Reflects negatively on applicant immigration success

**United Kingdom**	Yes¥	Yes	Not known	No

**Australia**	No	Yes	80%	No

**Israel**	Yes	†	--	--

**Jordan**	No	Yes	Not known	Residency permit not issued

**Additional Countries**				

**Norway**	Yes	No	--	--

**New Zealand**	Yes	No	--	--

**Sweden**	Yes	No	--	--

**Switzerland**	Yes	No	--	--

**Netherlands**	No	Yes	59%*	No

*compliance data for non refugee immigrant only[55]

† Migrants usually stay up to 1-2 years in the absorption centers

‡Follow up post arrival is done for persons with suspicious findings, but decentralized and the information is not collected systematically at central level

¥ Asylum seeker initial accommodation rather than refugee center

## Discussion

The majority of the countries participating in the study utilize some sort of immigration screening program. This may be related to the use of immigration as a policy of nation building as well as epidemiological evidence indicating a greater proportion of disease in foreign-born or migrant populations. In countries without routine screening programs the relatively low proportion of disease in the foreign-born along with low numbers of migrants may be reasons routine screening is not undertaken[30-34]. The primary objective of all TB screening programs is to detect active TB. Therefore, an important measure of the effectiveness of the program is the prevalence of active TB among those screened. While other indicators may be measured to assess TB immigration screening (migrant satisfaction and understanding of the program, adherence to the program by health professionals and migrants and the cost effectiveness of programs) they were not collected in this study. Our study demonstrates that the yield of active cases in terms of rates is several times the incidence of active disease in all of the host nations doing the screening where data were available (Table 3). It is important to note that several countries did not limit TB screening to applicants from high TB incidence countries. The yield of active pulmonary TB may be improved through more targeted application of screening practices, either based on countries of previous residence and/or risk factors for progression of LTBI to active disease[5,11,18,35-37]. Direct comparisons among countries must be made with caution as the characteristics and health determinants of persons screened vary by both by country of origin and by destination.

The location at which screening takes place varies between countries. Factors that determine screening location include domestic law, the annual number of immigrants admitted and cost of operations. For receiving countries, advantages of pre-departure screening include the prevention of the arrival of individuals with active or infectious disease. The major challenge in pre-departure screening noted across all countries is the difficulty of quality assurance of medical examinations and laboratory tests done overseas and fraudulent documents.

There is no standard determination of which migrant populations should be subject to immigration screening for TB. Some nations screen all applicants. Others only screen those seeking permanent residence or those staying for extended periods. Some nations screen on the basis of intended occupation. Some countries base the requirement for screening on the level of disease at the migrant's place of origin. In such countries, significant variability exists in the TB incidence rate of the source country, which will trigger an examination. Variations in the triggers for screening (origin, migrant class, occupation), migrant demography (source, age, duration of stay), nature of the immigration movement (refugee, asylum seeker, migrant worker etc.) make the comparison of national screening programs a challenging task. More detailed studies comparing disaggregated standardized information will be important in evaluating the impact and effect of national differences in screening.

As noted a limited number of nations are currently using an IGRA as a confirmatory test for all positive TSTs (two-step approach). The two-step approach improves the one-step approach (IGRA only) by preventing the loss of applicants who had a false negative IGRA result [38-40].

The expanded use of IGRAs suggested by recent studies does have limitations. Many applicants may be identified as having a positive test however many of them may not go for the follow up or agree to treatment. For countries that do pre-departure screening, logistical and operational aspects may be pose significant challenges to implementing the use of IGRAs in developing nations due to a lack of laboratory support and quality control.

All radiological screening programs are subject to interpretation error. Experience and the number of interpreters has been shown to affect interpretation. Standardized interpretation should be a goal of all screening programs using CXRs. Studies have [41] demonstrated that experience in reading CXRs for possible active TB among asylum seekers can play a significant role in the reproducibility of the reading. Although not done within an immigrant TB screening program, it has been suggested that [42] TB specialists had better accuracy in detecting TB related abnormalities. If films are digitized at each site and transmitted electronically to a central reading site, all interpretation can be done by a designated panel of TB radiology experts with an increase in the accuracy of diagnosing active and inactive PTB.

The greatest risk for the development of TB occurs in the first year after entering the host nation and then the risk decreases over time[43-45]. However, even though the risk diminishes over time, it is still higher than the rate among the destination country-born population even 10-20 years later [46-48]. Furthermore, the higher the TB burden in the country of origin, the higher the probability of disease following migration into the host country[49]. The attention on the identification of active disease at the time of immigration provided by all countries with screening programs will assist in the management of migration-related TB. However, without a corresponding program to manage LTBI and non-infectious TB in new arrivals, immigration screening will continue to have a limited impact on TB in the foreign born.

Origin, length of proposed stay and occupation all influenced screening requirements and varied among countries. Temporary residents can represent a significant source of TB[23,50].

International university/college students from high incidence countries can potentially be an important source of TB into the host nation. The literature suggest that foreign born students may contribute a significant amount of TB in a population and should thus be screened for LTBI after arrival at the school[50]. In the USA LTBI screening with TSTs of international students in one scholastic year found 35% of students to have a positive TST, of which 80% were successfully treated[51].

### Limitations of the study

Subtle cultural and political differences among countries are sometimes difficult to measure and thus report. Accuracy of the information reported is dependent on the participants. Effort was made to include only experts from each country. The accuracy of the information is also time sensitive and subject to change since it is always evolving. The survey was designed to compare particular aspects of the Canadian TB immigration screening program with other nations' programs. Other aspects that were present only in other countries may not have been included in the study.

### Common Goals - Partnerships between immigrant source and host countries

Several of the high immigration, low TB incidence countries did pre-departure screening in the countries of origin. Each receiving country had its own protocol and arrangements with (sometimes the same) laboratories, radiology services and examining physicians in the source countries. Program efficiency and effectiveness could result from the pooling of resources such as laboratory, radiology and examining physicians, (e.g., the practice of Australia and New Zealand sharing panel physicians). High standards of radiological and laboratory diagnosis are required by many screening countries. These services may be in short supply in high incidence regions. The support and improvement of TB services and management capacity in migrant source regions by migration destination countries which require immigration medical screening has both domestic and international benefit. More robust TB services in high TB incidence regions mutually supports TB prevention in migrants from those areas and in future host countries.

For example, as part of the U.S. implementation of their 2007 TB immigration screening requirements, the Centers for Disease Control and Prevention (CDC) helped develop laboratory and treatment programs to support their new culture and directly observed treatment requirements. Since implementation began, new TB laboratories (performing cultures and drug sensitivity testing) began to operate in Kenya, Mexico, Nepal, Thailand, and Vietnam. Existing laboratories have expanded greatly in the Dominican Republic and the Philippines. As part of this program, the CDC is trying to engage in-country TB organizations, such as National Tuberculosis Programs, so that the TB control infrastructure used for U.S. immigration applicants will also benefit the rest of the nation's population.

## Conclusions

As presented by others[3,52,53], immigration TB control needs to expand to a global level to achieve a significant decrease in the number of immigrants from high TB incidence countries who are found to have TB on immigration screening or to develop it soon after arrival. Host nations should invest in targeted TB programs in developing countries that form the bulk of the TB entering the country. Such an approach was shown to be cost effective in a U.S. based model[52]. This global approach to TB immigration must be based on a collaboration between TB screening immigration programs and the national TB programs in the countries of origin so that both immigrant source and receiving countries benefit.

In a globalized, mobile world, a case can be made for recommending that immigration medical screening should be a routine consideration for all national TB control programs. It is anticipated that immigration TB screening programs for high immigration low TB incidence countries will vary with national legislation, resource availability and public health risk management practices. Advances in medical science, diagnostic technology and therapeutic options continue to provide new considerations through which effectiveness of screening, both in terms of cost and health outcomes can be evaluated and improved. Technologies and resource allocation will vary by nation according to national priorities but targeting high risk populations remains an important feature of any screening program.

Improved screening of temporary residents and international university/college students may enhance existing national programs in some locations. Targeted screening of high risk migrants for LTBI with IGRA is a promising approach and further research is necessary. The digitization of CXRs which facilitates centralized interpretation by those with extensive TB experience is another area of interest in low incidence nations and may improve the accuracy of radiological screening. Advances in technology are important however to be effective these programs need to be integrated with domestic TB control programs and linked to international partners to ensure quality standards and coordinated patient care across borders are components of screening programs. Lastly, screening programs should adhere to international standards in terms of access to care and treatment, ethics and privacy data management.

## Competing interests

The authors declare that they have no competing interests.

## Authors' contributions

GGA designed the study, collected the data and did the analysis, and wrote the first draft, BG and EE contributed to the design, and made substantial revisions to the manuscript. KR, EA, DC, PD, CE, PH, IH, JJ, AM, MCP, DP, DS, ES, AT, ERV, BW contributed to acquisition of the data, read and revised the manuscript and gave approval of the final version to be published.

## Pre-publication history

The pre-publication history for this paper can be accessed here:

http://www.biomedcentral.com/1471-2334/11/3/prepub
